# Clinical-imaging fusion model for risk assessment of clinically significant prostate cancer

**DOI:** 10.3389/fonc.2026.1844810

**Published:** 2026-07-20

**Authors:** Yuyao Chen, Zhaole Yu, Jun Xu, Xuewei Kang, Liuke Liang, Jing He, Yunde Li, Qinghui Hu, Bao Feng, Pengmin Liu

**Affiliations:** 1 Department of Medical Imaging, Nanxishan Hospital of Guangxi Zhuang Autonomous Region, Gulin, China; 2 Laboratory of Intelligent Detection and Information Processing, Guilin University of Aerospace Technology, Guilin, China; 3 Department of Radiation Oncology, Affiliated Cancer Hospital of Guangxi Medical University and Cancer Institute of Guangxi Zhuang Autonomous Region, Nanning, Guangxi, China; 4 School of Computer Science and Engineering, Guilin University of Aerospace Technology, Guilin, China; 5 Cancer Center, Shanxi Bethune Hospital, Shanxi Academy of Medical Sciences, Tongji Shanxi Hospital, Third Hospital of Shanxi Medical University, Taiyuan, China

**Keywords:** clinically significant prostate cancer, prostate magnetic resonance imaging, risk assessment, transfer learning, vision foundation models

## Abstract

**Background:**

Prostate cancer is the second most commonly diagnosed malignancy among men worldwide. Its biological heterogeneity challenges conventional imaging-based risk assessment and complicates biopsy decision-making. Although advanced artificial intelligence may improve risk stratification, medical imaging applications are often limited by small sample sizes. Vision foundation models, with strong transferability in data-constrained settings, offer a promising approach to improving prostate cancer risk assessment, guiding personalized treatment, and reducing overdiagnosis.

**Methods:**

A robust transfer learning framework based on prostate magnetic resonance imaging (MRI), termed robust transfer learning model (RTLM), was developed to enable the non-invasive risk assessment of clinically significant prostate cancer (csPCa). RTLM employs a feature-matching transfer strategy to adaptively capture task-relevant abstract knowledge from vision foundation models, thereby enhancing the feature representation capability and robustness of convolutional neural networks. Model performance was evaluated on csPCa MRI data, and a multi-task evaluation was conducted on PD-L1 expression prediction in patients with non-small cell lung cancer (NSCLC) to assess the generalizability of the proposed framework. Subsequently, features extracted by the RTLM were used to construct a deep learning signature (DLS), which was then integrated with key clinical variables, including age, serum total PSA level, PSA density, prostate volume, and PI-RADS score, to build the clinical-imaging fusion model (CIFM) for csPCa risk assessment. Diagnostic performance was evaluated using the area under the curve (AUC), decision curve analysis (DCA), integrated discrimination improvement (IDI), and net reclassification improvement (NRI).

**Results:**

The CIFM achieved AUCs of 0.918, 0.890, 0.828, and 0.852 in the training cohort (n = 585), test cohort (n = 310), and two external validation cohorts (n = 510 and n = 94), respectively. Compared with the clinical model and the RTLM, CIFM showed significant improvements in both IDI and NRI (all p < 0.05). Decision curve analysis further demonstrated that CIFM provided greater net clinical benefit.

**Conclusion:**

By effectively leveraging knowledge representations from vision foundation models, RTLM enhances the feature learning capability and robustness of CNNs in small-sample medical imaging tasks. The CIFM, which integrates clinical indicators, demonstrates good accuracy in csPCa risk assessment, suggesting potential generalizability.

## 1 Introduction

Prostate cancer is the second most commonly diagnosed malignancy among men worldwide and ranks first in cancer incidence among men in approximately two-thirds of countries (1). However, prostate cancer exhibits marked heterogeneity: some tumors are highly aggressive, whereas others progress slowly or even remain asymptomatic (2). To identify high-risk patients, Epstein et al. proposed the concept of clinically significant prostate cancer (csPCa) in 1994 and established diagnostic criteria including tumor volume, Gleason score, and extraprostatic extension (3). With advances in medical imaging technology, prostate MRI has become increasingly important for the detection of csPCa. The widely used PI-RADS scoring system assigns lesions a score from 1 to 5 to estimate the likelihood of csPCa, with higher scores indicating a greater risk of malignancy, and it is commonly used to guide biopsy decisions (4, 5). However, in clinical practice, PI-RADS still suffers from limitations such as relatively low positive predictive value (6), operator-dependent specificity and inter-reader consistency (7), and a considerable rate of missed or misdiagnosed cases (8), indicating that tumor-related information contained in MRI images has not yet been fully exploited. Therefore, effectively capturing and utilizing tumor feature information from MRI is crucial for prostate cancer risk assessment.

In recent years, deep learning has achieved remarkable progress in medical image analysis (9–11). Convolutional neural networks (CNNs), owing to their strong local feature learning capabilities, have been widely applied to tumor detection, grading, and prognosis prediction (12, 13). However, CNN-based models mainly rely on local convolutional receptive fields and require sufficient task-specific training data; therefore, their feature representation and generalization may be limited when applied to small and heterogeneous medical imaging datasets (14). Recent transformer- and attention-based studies have attempted to address these limitations by modeling long-range dependencies, emphasizing informative image regions, and improving model interpretability. For example, Mir et al. proposed an explainable vision transformer framework for histopathological image analysis and combined multiple interpretation strategies, demonstrating the potential of transformer architectures to improve both feature representation and transparency (15). Similarly, Mir and Rizvi developed an EfficientNet-CBAM-based framework for brain tumor MRI classification, showing that channel and spatial attention mechanisms can strengthen lesion-related feature extraction and model explainability (16).

Nevertheless, these studies mainly focus on image-only classification and interpretability in specific disease settings; they do not directly address how to selectively transfer knowledge from large vision foundation models to small prostate MRI datasets, nor how to integrate imaging-derived features with individualized clinical information for csPCa risk assessment. In addition, conventional clinical prediction models are relatively interpretable but cannot fully exploit sub-visual MRI heterogeneity, whereas image-only deep learning models may overlook established clinical risk factors. In contrast, vision foundation models (VFMs) pretrained on large-scale datasets demonstrate superior semantic representation capability in general image understanding, providing a promising new avenue for medical image analysis (17). However, their large parameter scale and high training cost make end-to-end fine-tuning on limited medical data prone to overfitting and computationally expensive. Consequently, transfer learning is commonly adopted to leverage the general knowledge of vision foundation models for improving medical imaging tasks. Yet, the substantial distribution gap between natural images and medical images means that naive transfer strategies may lead to negative transfer, thereby degrading the model’s ability to identify key lesion features (18, 19).

To address these challenges, this study proposes a robust transfer learning model based on vision foundation models (RTLM) to fully exploit the general knowledge of VFMs while mitigating the adverse effects of negative transfer. RTLM introduces a feature-matching transfer mechanism that adaptively determines which knowledge should be transferred from the vision foundation model and to which layers of the CNN (20). Specifically, two feature-matching transfer networks are constructed: one to identify the layer-wise correspondence between the vision foundation model and the CNN, and the other to select the specific features to be transferred as well as the degree of transfer. Through these networks, the feature learning process of the local CNN is effectively constrained and guided, enabling task-specific knowledge transfer and thereby enhancing the feature representation capability and robustness of the CNN.

Furthermore, considering that csPCa risk assessment depends not only on imaging manifestations but also on individualized clinical information, this study builds a deep learning signature (DLS) on the basis of RTLM and integrates it with key clinical variables, including age, serum total PSA, PSA density, prostate volume, and PI-RADS score, to establish a clinical-imaging fusion model (Clinical-Imaging Fusion Model, CIFM). The DLS focuses on characterizing latent structural features and imaging heterogeneity of lesions, whereas clinical variables reflect patients’ physiological status and underlying disease risk; their complementarity is expected to enhance the comprehensive representation of csPCa, thereby enabling more accurate non-invasive risk stratification and decision support. This study evaluates the performance of RTLM and CIFM in csPCa risk assessment using metrics including the area under the curve (AUC), decision curve analysis (DCA), integrated discrimination improvement (IDI), and net reclassification improvement (NRI), to assess their clinical net benefit and risk reclassification value. In addition, cross-task validation is conducted to further verify the generalization capability of RTLM.

## 2 Materials and methods

### 2.1 Prostate MRI image assessment

As the prostate MRI diagnostic and evaluation criteria varied across the hospitals/institutions from which the data were obtained (e.g., some institutions adopted a five-point scoring system similar to PI-RADS but with different scoring details), and evaluation standards also differed across years within the same institutions (e.g., PI-RADS v1, v2, and v2.1 were used in different periods), all prostate MRI examinations in this study were re-evaluated according to a unified standard. Image assessment was performed by two radiologists with 5 and 16 years of experience in prostate MRI interpretation, respectively (Radiologist 1 and Radiologist 2), who were classified as “basic” and “expert” level radiologists according to the consensus statement of the European Society of Urogenital Radiology and the EAU Section of Urogenital Imaging.The inter-reader agreement for PI-RADS scoring between the two radiologists was excellent, with a quadratic weighted κ of 0.964 (P < 0.001;**Supplementary Table 6**).

Prostate MRI evaluation was conducted with reference to PI-RADS v2.1 (21). Suspected prostate lesions were scored using a five-point scale. Prostate volume was calculated using the ellipsoid formula (maximum anteroposterior diameter × maximum transverse diameter × maximum craniocaudal diameter × 0.52). The final prostate size measurements were obtained by averaging the values measured by the two radiologists. When multiple suspicious lesions were identified in the prostate, the lesion with the highest likelihood of malignancy (i.e., the highest score) was selected as the overall score for that case. In cases of disagreement between the two radiologists, a third senior radiologist with 25 years of MRI diagnostic experience made the final decision. Patients were dichotomized into csPCa and ncsPCa; the ncsPCa group comprised benign cases and non-clinically significant prostate cancer.

### 2.2 Patients

This study was a retrospective observational study. Ethical approval was obtained from the hospital ethics committee, and the requirement for informed consent was waived. For cases obtained from public datasets, the data providers authorized their use for non-profit academic research, and ethical approval and informed consent waivers had already been granted by the ethics committees of the participating institutions.

The cases included in this study were derived from four sources: 1. The public training and development datasets of the PI-CAI Challenge (22); 2. The Prostate-MRI-US-Biopsy public dataset (23); 3. Patients who visited Nanxishan Hospital for suspected prostate disease and underwent examinations; 4. Data from patients with non-small cell lung cancer (NSCLC).

The public training and development dataset of the PI-CAI Challenge consisted of patients who underwent examinations for suspected prostate cancer between 2012 and 2021 at one of three centers in the Netherlands (Radboud University Medical Center, Ziekenhuisgroep Twente, and Prostaat Centrum Noord-Nederland). The inclusion criteria specified by the data providers were: (1) elevated serum PSA levels and/or abnormal digital rectal examination findings; and (2) availability of prostate MRI examinations. The exclusion criteria were: (1) refusal or lack of consent for reuse of clinical data; (2) prior prostate treatment; (3) a history of pathologically confirmed csPCa; (4) severe MRI artifacts; and (5) csPCa identified on pathology that could not be localized on MRI. A total of 895 patients were included.

The Prostate-MRI-US-Biopsy public dataset included patients who underwent prostate biopsy at the UCLA Clark Urological Center between 2006 and 2011 due to elevated serum PSA levels and/or suspicious imaging findings. The dataset initially contained 1,151 cases. Excluded cases included those with missing images (n = 330), insufficient image quality (n = 31), postoperative prostate changes visible on imaging (n = 19), a prior pathological diagnosis of prostate cancer (n = 15), an interval of more than three months between biopsy and MRI (n = 80), missing age information (n = 4), missing serum PSA results (n = 2), or missing Gleason scores (n = 1). After applying these criteria, 669 cases remained. Among them, additional cases were further excluded due to image quality issues, resulting in a final cohort of 510 patients for model validation.

For the Nanxishan Hospital dataset, cases were collected between October 2021 and September 2025. The inclusion criteria were: (1) pathologically confirmed prostate cancer; (2) no prior radical prostatectomy, radiotherapy, endocrine therapy, chemotherapy, or other treatments targeting prostate lesions before prostate MRI and biopsy; and (3) prostate MRI performed within two weeks before biopsy. The exclusion criteria were: (1) a previous history of prostate cancer; (2) excessive MRI artifacts affecting evaluation; and (3) incomplete prostate imaging or clinical data. A total of 94 patients were included.

For the NSCLC cohort, PD-L1 expression data were collected retrospectively from January 2018 to April 2021. The inclusion criteria were: (1) histopathologically confirmed lung cancer, including different types of non-small cell lung carcinoma, after biopsy or surgical resection; (2) availability of chest CT images prior to biopsy or resection; and (3) quantitative results of PD-L1 expression. The exclusion criteria were: (1) absence of contrast-enhanced CT images; (2) use of immunohistochemical antibodies other than Dako 22C3 for PD-L1 detection; and (3) poor CT image quality.

The PI-CAI dataset was randomly divided into training and test cohorts for model training and validation. The Prostate-MRI-US-Biopsy dataset was used as external validation cohort 1, the Nanxishan Hospital dataset as external validation cohort 2, and the NSCLC dataset for additional cross-task validation. The distribution of patients in the prostate datasets is summarized in **Table 1**.

**Table 1:** Patient information.

Clinical factors	Training cohort(n=585)		P value	Test cohort(n=310)		P value	External validation cohort 1(n=510)		P value	External validation cohort 2(n=94)		P value
	ncsPCa (n=337)	csPCa (n=248)		ncsPCa (n=182)	csPCa (n=128)		ncsPCa(n=248)	csPCa(n=262)		ncsPCa(n=28)	csPCa(n=66)	
Age (years, mean ± SD)	65.66 ± 6.995	68.16 ± 6.741	<0.001	65.41 ± 6.769	66.43 ± 7.573	0.173	63.69 ± 6.892	66.79 ± 7.562	<0.001	71.68 ± 10.089	73.09 ± 9.524	0.520
Prostate volume (cm³; mean ± SD)	64.42 ± 33.541	53.48 ± 25.284	<0.001	68.65 ± 35.791	47.003 ± 21.868	<0.001	60.399 ± 29.636	47.390 ± 24.728	<0.001	66.958 ± 44.510	55.199 ± 29.244	<0.001
Serum total PSA (ng/ml; mean ± SD)	11.70 ± 15.332	16.49 ± 21.025	<0.001	10.091 ± 9.753	15.295 ± 14.805	<0.001	7.568 ± 6.436	11.798 ± 14.260	<0.001	25.138 ± 26.375	263.792 ± 584.673	0.201
PSA density (mean ± SD)	0.199 ± 0.252	0.337 ± 0.419	<0.001	0.163 ± 0.154	0.361 ± 0.349	<0.001	0.145 ± 0.176	0.285 ± 0.329	<0.001	0.412 ± 0.451	4.695 ± 10.975	<0.001
PI-RADS score												
1-2	35	1	<0.001	14	0	0.001	25	7	0.001	0	1	0.513
3-5	302	247		168	128	223	255	28	65

The normality of continuous variables was assessed before statistical comparison. Only age in the test cohort and external-validation cohort 2 met the assumptions for parametric testing and was further assessed for homogeneity of variance using Levene’s test; these comparisons were performed using the independent-samples t test. All other continuous variables showed non-normal distributions and were compared using the Mann–Whitney U test. Categorical variables were compared using the chi-square test or Fisher’s exact test, as appropriate. A two-sided P value <0.05 was considered statistically significant.

### 2.3 Delineation of regions of interest in MRI images

**Figure 1:**
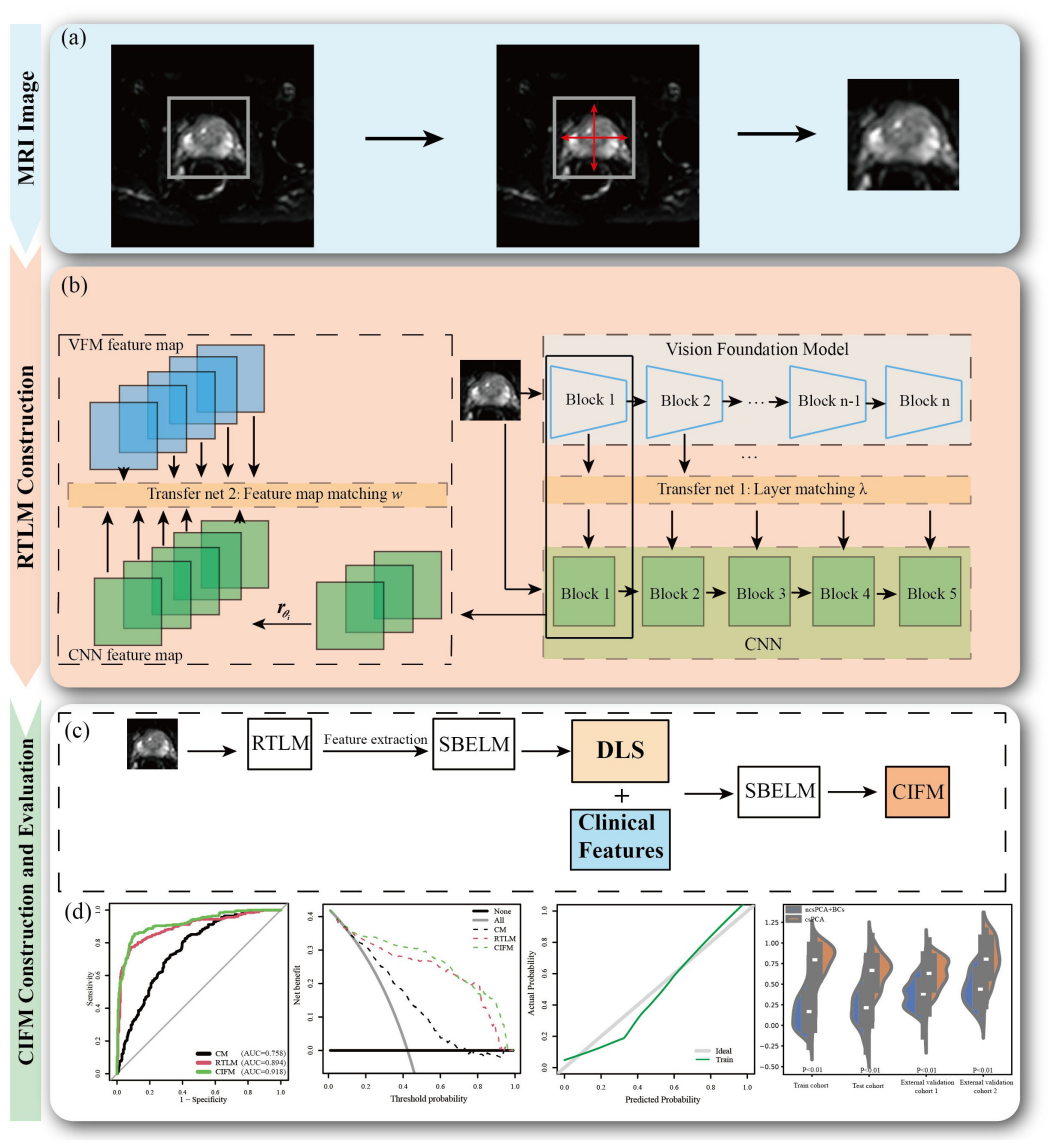
Overall workflow of the proposed method. **(A)** MRI preprocessing and extraction of the prostate region of interest. **(B)** Construction of the robust transfer learning model (RTLM), including feature-map matching and layer-wise matching between the vision foundation model and the convolutional neural network. **(C)** Construction of the deep learning signature (DLS) and the clinical-imaging fusion model (CIFM) by integrating RTLM-derived imaging features with clinical variables. **(D)** Evaluation of the proposed models using receiver operating characteristic curves, decision curve analysis, calibration curves, and predicted-score distribution plots.

Three radiologists with extensive experience in prostate MRI interpretation delineated the entire prostate along with adjacent surrounding tissues on diffusion-weighted images (DWI) for each case, and the center coordinates of the prostate were obtained within the delineated region. Using this center point as the reference, the region was expanded by 64 pixels in the superior, inferior, left, and right directions to ensure that the entire prostate was included within the region of interest (ROI), resulting in images of size 128×128 pixels. To meet the input size requirements of the model, all images were subsequently resized to 224×224 pixels, as illustrated in **Figure 1A**. Because prostate MRI is volumetric, the ROI extraction procedure was applied slice by slice to all available prostate-containing slices. The 2D ROI from each slice was then used for subsequent feature extraction, and the slice-level features from the same patient were arranged according to their anatomical order and integrated into a patient-level representation before classification. This strategy allowed volumetric information from the slice stack to be incorporated at the feature level while avoiding direct 3D resampling, padding, or slice truncation across multicenter datasets with heterogeneous slice numbers, slice thicknesses, and prostate coverage.

### 2.4 Construction of the clinical model

In csPCa risk aThree radiologists with extensive experience in prostate MRI interpretation delineated the entire prostate along with adjacent surrounding tissues on diffusion-weighted images (DWI) for each case, and the center coordinates of the prostate were obtained within the delineated region. Using this center point as the reference, the region was expanded by 64 pixels in the superior, inferior, left, and right directions to ensure that the entire prostate was included within the region of interest (ROI), resulting in images of size 128 × 128 pixels. To meet the input size requirements of the model, all images were subsequently resized to 224 × 224 pixels, as illustrated in **Figure 1A**.

For model construction, the 2D ROI from each slice was processed using a shared feature extractor. The extracted features from all available slices of the same patient were then arranged according to their anatomical order and concatenated into a patient-level representation before classification. This feature-level slice aggregation allowed the model to incorporate volumetric information from the slice stack while avoiding direct 3D resampling, interpolation, padding, or slice truncation, which could introduce additional variability in multicenter datasets with different slice thicknesses, inter-slice spacing, and prostate coverage.

In csPCa risk assessment, the selected clinical indicators have well-established clinical relevance. Age is a fundamental risk factor for prostate cancer; prostate volume influences the interpretation of PSA levels and helps distinguish false positives caused by benign prostatic hyperplasia; serum total PSA is the most commonly used screening marker; PSA density more effectively reflects tumor burden after adjusting for prostate volume; and the PI-RADS score, as a structured MRI-based imaging system, directly assesses lesion suspicion.

First, statistical analyses were performed on patients’ clinical characteristics, including age, prostate volume, serum total PSA level, PSA density, and PI-RADS score. For continuous variables, normality was assessed separately in the ncsPCa and csPCa groups within each cohort using the Shapiro–Wilk test. For variables that were normally distributed in both comparison groups, homogeneity of variance was further assessed using Levene’s test. Normally distributed variables with homogeneous variances were compared using the independent-samples t test, whereas normally distributed variables with unequal variances were compared using Welch’s t test. Variables that were non-normally distributed in at least one comparison group were compared using the Mann–Whitney U test. Categorical variables were compared using the chi-square test or Fisher’s exact test, as appropriate. Clinical indicators with statistical significance in the training cohort (P < 0.05) were selected as candidate variables. Subsequently, five variables—age, prostate volume, serum total PSA level, PSA density, and PI-RADS score—were incorporated into a Sparse Bayesian Extreme Learning Machine to construct the clinical model (CM).

### 2.5 Robust transfer learning model based on vision foundation models

While vision foundation models (e.g., DINOv2-Base) exhibit robust generalized feature representation, direct application to medical imaging often leads to negative transfer. This is primarily due to the significant domain gap between natural images and the subtle, heterogeneous textures characteristic of prostate MRI. To address this issue, we propose RTLM, which employs selective feature-matching transfer to adaptively determine which knowledge to transfer, to which layers, and with what transfer intensity, thereby improving the robustness and generalization of CNN models in capturing csPCa-specific imaging biomarkers (**Figure 1B**).

RTLM constructs two cooperative feature-matching transfer networks. One network learns the layer-wise correspondence between the vision foundation model and the CNN and adjusts the transfer weights across layers, while the other assigns transfer weights at the feature level for cross-model feature alignment. This strategy strengthens features that are critical for characterizing prostate lesions (e.g., tumor margins and tissue heterogeneity) while suppressing redundant or unsuitable generic features. During training, the transfer constraint is jointly optimized as a regularization term together with the supervised loss of the CNN, enabling the CNN to maintain local discriminative capability while receiving robust representational guidance from the vision foundation model. After transfer training, features are extracted from the optimized CNN to construct the DLS, serving as a quantitative, sub-visual imaging surrogate for csPCa risk. Detailed algorithmic descriptions are provided in Supplementary Material 1. Unlike conventional transfer learning, which usually transfers pretrained weights without explicitly measuring local feature suitability, RTLM uses feature matching together with adaptive layer- and feature-level weights to control both the source and intensity of transferred knowledge.

### 2.6 Construction of the clinical-imaging fusion model

In routine clinical practice, the risk assessment of csPCa relies on a multidisciplinary approach rather than imaging alone. To fully integrate patients’ clinical indicators with deep learning-based imaging features and mirror this comprehensive clinical workflow, the CIFM was developed.

Statistical comparisons were first performed for candidate clinical variables, including age, serum total PSA level, prostate volume, PSA density, and PI-RADS score. Continuous variables were compared using independent-samples t tests or Mann–Whitney U tests according to their distribution, while categorical variables were compared using chi-square tests or Fisher’s exact tests, as appropriate. Variables significantly associated with csPCa in the training cohort (P < 0.05) were selected for model construction. The selected clinical variables were then combined with the DLS and incorporated into the classifier, with csPCa status coded as 0 = ncsPCa and 1 = csPCa as the dependent variable. The final CIFM consisted of the DLS, age, PSA density, prostate volume, and PI-RADS score for comprehensive prediction of csPCa risk. The construction of the DLS and CIFM is illustrated in **Figure 1C**.

### 2.7 Statistical analysis

To comprehensively evaluate the predictive performance of each model, multiple metrics were calculated for the CM, RTLM, and the CIFM on the training cohort, test cohort, and external validation cohorts, including the AUC, sensitivity, specificity, accuracy, positive predictive value (PPV), and negative predictive value (NPV).

To further compare the discriminative ability and classification improvement among different models, IDI and NRI were used to quantitatively assess the performance gains of CIFM over the clinical model and RTLM, thereby evaluating its superiority in risk reclassification. In this study, we assessed NRI using the continuous NRI approach. Therefore, no predefined risk-category threshold was required; the analysis evaluated whether predicted risks moved in the correct direction for patients with and without csPCa when CIFM was compared with the clinical model or RTLM.

In addition, DCA was performed to assess the net clinical benefit of CIFM across different decision thresholds, thereby validating its clinical utility. Calibration curves were also plotted to examine the agreement between predicted probabilities and observed outcomes, enabling evaluation of the calibration performance of the model. The overall model evaluation framework is summarized in **Figure 1D**.

### 2.8 Experiment settings

In this study, the vision foundation model used was DINOv2 (17), while the local model adopted a ResNet18 architecture (24). The ResNet18 model parameters were initialized using pretrained weights from the ImageNet dataset. The initial learning rate was set to 0.001, with a batch size of 8 images of size 224 × 224 per iteration. The optimizer used was stochastic gradient descent (SGD) with momentum, where the momentum was set to 0.9 and the weight decay to 0.0001.

All experiments were conducted on a workstation equipped with an Intel(R) Core(TM) i9-9900K CPU (3.50 GHz), an NVIDIA TITAN A6000 GPU, and 48 GB of RAM, running the Windows 11 operating system. Python was used to build and train the deep learning framework, and SPSS software was used for statistical analysis.

## 3 Results

### 3.1 Clinical characteristics and construction of the clinical model

Univariate statistical analyses identified patient age, serum total PSA level, PSA density, PI-RADS score, and prostate volume as the major clinical features associated with csPCa. These variables were used to construct the CM. **Table 1** summarizes the comparisons of clinical characteristics between the csPCa group and the ncsPCa group across all datasets. The results showed that, in the training cohort, significant differences were observed between the two groups in terms of age, prostate volume, serum total PSA level, PSA density, and PI-RADS score (all P < 0.001), indicating that these clinical variables were statistically meaningful for distinguishing csPCa from ncsPCa.

In the training cohort, the clinical model achieved an AUC of 0.758 (95% CI: 0.7193-0.7960), with a sensitivity of 0.827 and a specificity of 0.585. In the test set, the AUC was 0.742 (95% CI: 0.6866-0.7974), with a sensitivity of 0.734 and a specificity of 0.604, demonstrating stable model performance. In external validation cohorts 1 and 2, the AUCs were 0.726 (95% CI: 0.6821-0.7693) and 0.738 (95% CI: 0.6312-0.8440), respectively, indicating good generalization capability.

### 3.2 Diagnostic performance of RTLM

In constructing the deep learning model, this study leveraged the powerful feature representation capability of a vision foundation model (DINOv2-Base, 86M parameters) and implemented knowledge transfer and feature guidance through a robust feature transfer network. This network adaptively selects the representations most relevant to the target task from the vision foundation model and transfers them to specific layers of the local model, thereby enhancing attention to critical imaging features and improving discriminative performance. After model training, intermediate features were extracted from multiple layers to build a multi-level feature representation, which was then fed into the classifier to generate predicted risk scores, yielding the quantitative DLS. This robust transfer strategy based on vision foundation models effectively mitigated overfitting under small-sample conditions and improved the model’s ability to discriminate between csPCa and ncsPC.

Across the training cohort, test cohort, and two external validation cohorts, the deep learning model achieved AUCs of 0.894 (95% CI: 0.8666-0.9221), 0.848 (95% CI: 0.8065-0.8886), 0.815 (95% CI: 0.7802-0.8504), and 0.827 (95% CI: 0.7291-0.9257), respectively. Compared with the CM, RTLM consistently yielded higher AUCs as well as improved sensitivity and specificity across all datasets, reflecting stronger discriminative performance and generalization capability. These results indicate that the RTLM design based on deep MRI features provides superior diagnostic performance and stability for csPCa risk prediction.

Furthermore, to validate the effectiveness and transferability of RTLM, cross-task validation was performed for the prediction of PD-L1 expression in patients with NSCLC. The clinical information of the NSCLC dataset and the distribution of PD-L1 expression are shown in **Supplementary Table 1**. In this task, RTLM achieved AUCs of 0.892 and 0.877 in the training and test cohorts, respectively. In the test cohort, the model demonstrated favorable performance, with a sensitivity of 0.815 (22/27), specificity of 0.833 (15/18), overall accuracy of 0.822 (37/45), positive predictive value (PPV) of 0.880 (22/25), and negative predictive value (NPV) of 0.750 (15/20). Meanwhile, to provide a clinical-reference comparison for the NSCLC PD-L1 prediction task, we incorporated the available clinical indicators in the NSCLC cohort, including age, sex, pathological type, and tumor stage, to construct an NSCLC-specific clinical model. This clinical model was newly trained and validated within the NSCLC task rather than being transferred from the prostate cancer task. To maintain methodological consistency across tasks, the NSCLC-specific clinical model was constructed using the same classifier as that used in the prostate cancer task. The model parameters of the clinical model are presented in **Supplementary Table 2**. Compared with the clinical model, RTLM achieved superior overall performance, with detailed diagnostic results presented in **Supplementary Table 3**. These findings further confirm that RTLM can effectively transfer task-relevant knowledge from vision foundation models, enhance the sensitivity and discriminative power of CNNs for imaging features across different diseases, and demonstrate good cross-task generalization and scalability.

### 3.3 Validation and evaluation of CIFM

To validate the effectiveness of CIFM for csPCa risk prediction, we compared the diagnostic performance of the clinical model (CM), RTLM, PICG2 (25), Z-SSMNet (26), and CIFM across the training cohort, test cohort, and two independent external validation cohorts. Among them, PICG2 and Z-SSMNet were derived from related algorithms developed on the PI-CAI and MRI-US datasets, respectively. By incorporating these comparative models, we aimed to provide a more comprehensive evaluation of the discriminative performance, robustness, and generalizability of CIFM for csPCa risk prediction. Because the official PI-CAI leaderboard is based on a hidden evaluation setting and some submitted methods do not provide reproducible model weights or patient-level predictions, we focused on comparable methods that could be evaluated under the available datasets and a consistent evaluation protocol.

**Figure 2:**
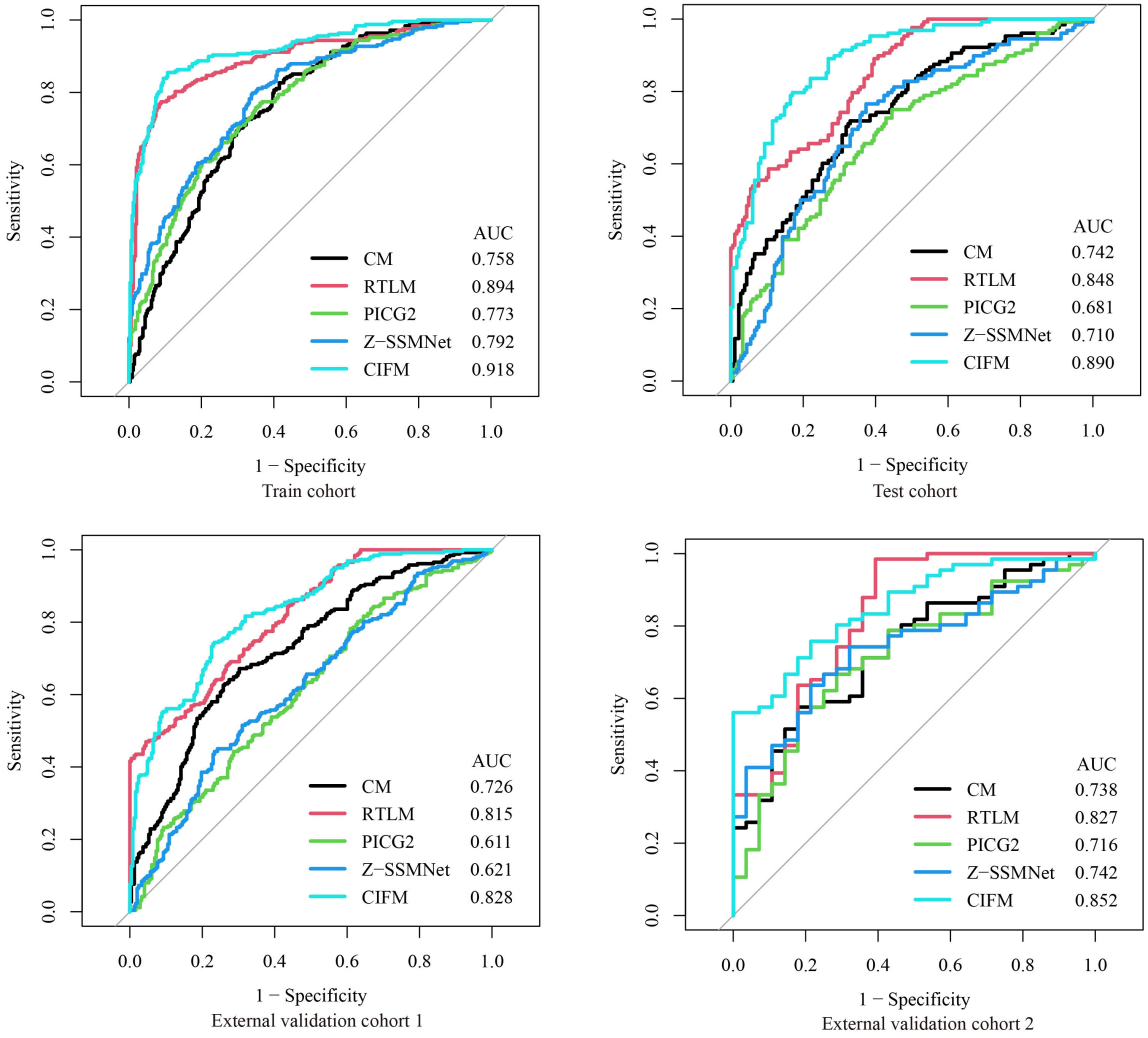
Five models’ ROC curves on the training cohort, test cohort, and two external validation cohorts. CM, Clinical Model; RTLM, Robust Transfer Learning Model; PICG2, PI-RADS Clinical Guidelines; Z-SSMNet, Zonal-aware Self-supervised Mesh Network; CIFM, Clinical-Imaging Fusion Model.

**Figure 3:**
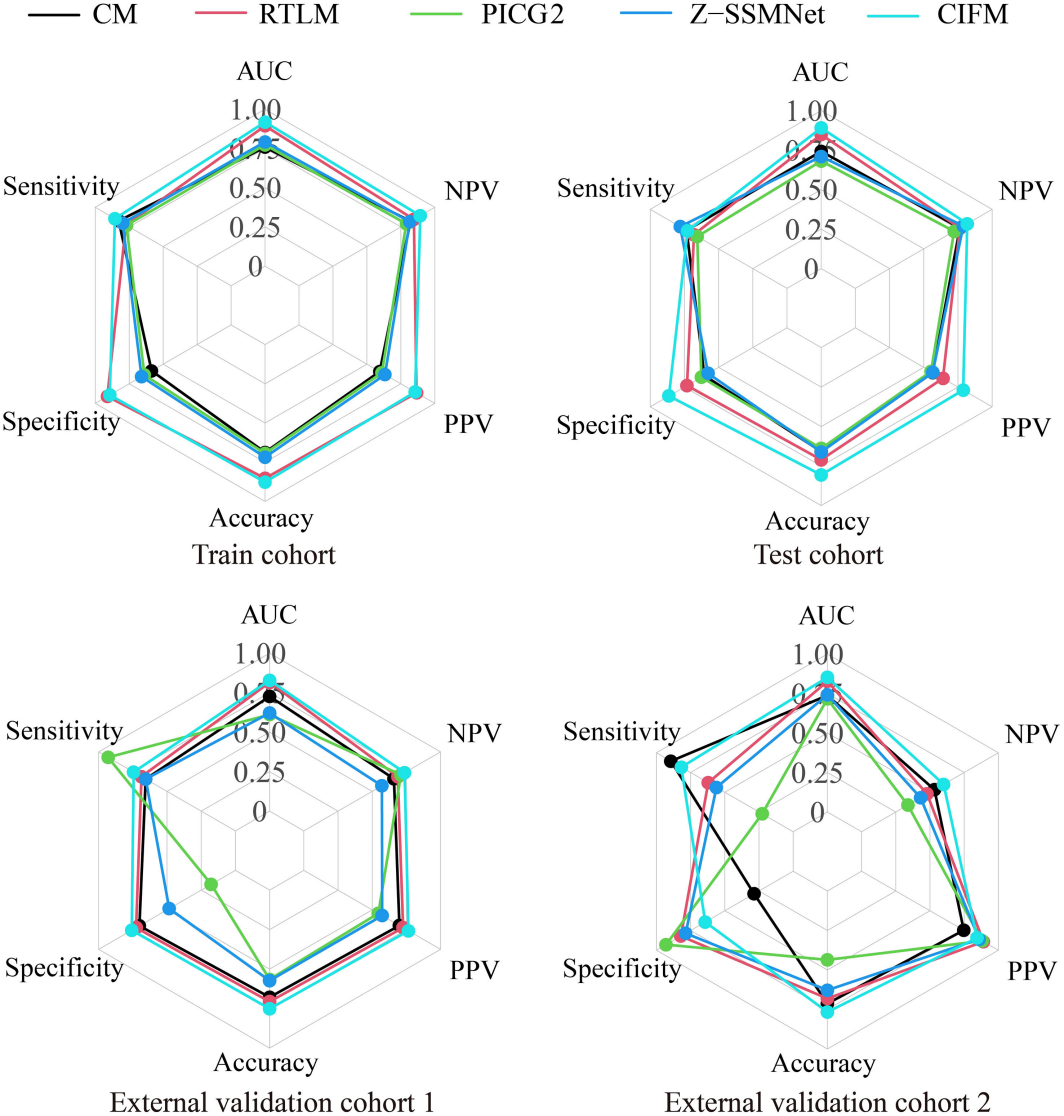
Radar plots of the five models on the training cohort, test cohort, and two external validation cohorts.

In the training cohort (n = 585), CIFM achieved the highest AUC of 0.918 (95% CI: 0.8952-0.9417), outperforming CM (0.758) and RTLM (0.894). CIFM also demonstrated balanced sensitivity (0.855) and specificity (0.893), indicating strong overall discriminative ability and stability. In the test set (n = 310), CIFM achieved an AUC of 0.890 (95% CI: 0.8547-0.9250), again surpassing CM (0.742) and RTLM (0.848). In external validation cohorts 1 (n = 510) and 2 (n = 94), the AUCs of CIFM were 0.828 (95% CI: 0.7936-0.8627) and 0.852 (95% CI: 0.7760-0.9285), respectively, both higher than those of the other models, further confirming its robustness and generalization across centers. Detailed diagnostic performance metrics are provided in **Table 2**, and **Figure 2** illustrates the ROC curves of the five models across all datasets. As shown in **Figure 3**, CIFM achieved the highest overall discriminative performance in all cohorts. To provide a direct comparison of model complexity, the number of parameters of CM, RTLM, and CIFM is reported in **Supplementary Table 5**. Compared with RTLM, CIFM increased the number of parameters only from 14,994,465 to 14,994,618, corresponding to an additional 153 parameters (approximately 0.0010%). This small increase suggests that the performance improvement of CIFM was mainly related to the complementary clinical information rather than a substantial increase in model complexity.

**Table 2:** The diagnostic performance of the five models on the training cohort, test cohort, and two external validation cohorts.

Cohorts	Models	AUC (95% CI)	Sensitivity	Specificity	Accuracy	PPV	NPV
Training (n=585)	PICG2	0.773(0.7359-0.8109)	0.770 (191/248)	0.638 (215/337)	0.694 (406/585)	0.610 (191/313)	0.790 (215/272)
	Z-SSMNet	0.792(0.7554-0.8282)	0.798 (198/248)	0.659 (222/337)	0.718 (420/585)	0.633 (198/313)	0.816 (222/272)
	CM	0.758(0.7193-0.7960)	0.827(205/248)	0.585(197/337)	0.687(402/585)	0.594(205/345)	0.821(197/240)
	RTLM	0.894(0.8666-0.9221)	0.774(192/248)	0.911(307/337)	0.853(499/585)	0.865(192/222)	0.846(307/363)
	CIFM	0.918(0.8952-0.9417)	0.855(212/248)	0.893(301/337)	0.877(513/585)	0.855(212/248)	0.893(301/337)
Test(n=310)	PICG2	0.681(0.6207-0.7413)	0.656 (84/128)	0.626 (114/182)	0.639 (198/310)	0.553 (84/152)	0.722 (114/158)
	Z-SSMNet	0.710(0.6517-0.7690)	0.781 (100/128)	0.577 (105/182)	0.661 (205/310)	0.565 (100/177)	0.789 (105/133)
	CM	0.742(0.6866-0.7974)	0.734(94/128)	0.604(110/182)	0.658(204/310)	0.566(94/166)	0.764(110/144)
	RTLM	0.848(0.8065-0.8886)	0.680(87/128)	0.731(133/182)	0.710(220/310)	0.640(87/136)	0.764(133/174)
	CIFM	0.890(0.8547-0.9250)	0.727(93/128)	0.863(157/182)	0.806(250/310)	0.788(93/118)	0.818(157/192)
External validation Cohort 1(n=510)	PICG2	0.611(0.5620-0.6595)	0.931 (244/262)	0.177 (44/248)	0.565 (288/510)	0.545 (244/448)	0.710 (44/62)
	Z-SSMNet	0.621(0.5725-0.6694)	0.656 (172/262)	0.484 (120/248)	0.573 (292/510)	0.573 (172/300)	0.571 (120/210)
	CM	0.726(0.6821-0.7693)	0.656(172/262)	0.702(174/248)	0.678(346/510)	0.699(172/246)	0.659(174/264)
	RTLM	0.815(0.7802-0.8504)	0.687(180/262)	0.726(180/248)	0.706(360/510)	0.726(180/248)	0.687(180/262)
	CIFM	0.828(0.7936-0.8627)	0.744(195/262)	0.758(188/248)	0.751(383/510)	0.765(195/255)	0.737(188/255)
External validation Cohort 2(n=94)	PICG2	0.716(0.6043-0.8275)	0.227 (15/66)	0.929 (26/28)	0.436 (41/94)	0.882 (15/17)	0.338 (26/77)
	Z-SSMNet	0.742(0.6413-0.8436)	0.561 (37/66)	0.786 (22/28)	0.628 (59/94)	0.860 (37/43)	0.431 (22/51)
	CM	0.738(0.6312-0.8440)	0.894(59/66)	0.286(8/28)	0.713(67/94)	0.747(59/79)	0.533(8/15)
	RTLM	0.827(0.7291-0.9257)	0.621(41/66)	0.821(23/28)	0.681(64/94)	0.891(41/46)	0.479(23/48)
	CIFM	0.852(0.7760-0.9285)	0.818(54/66)	0.643(18/28)	0.766(72/94)	0.844(54/64)	0.600(18/30)

**Figure 4:**
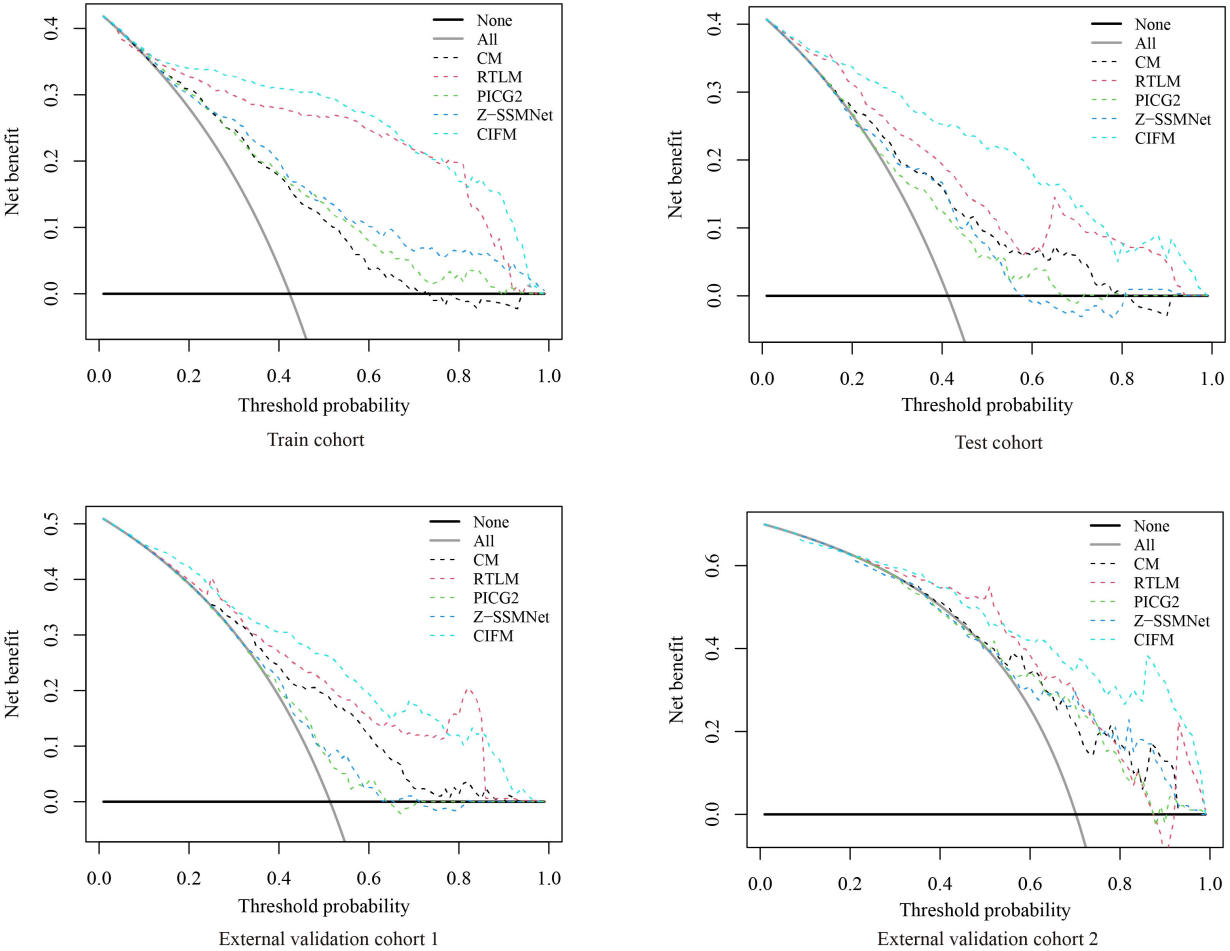
DCA curves of the five models on the training cohort, test cohort, and two external validation cohorts.

**Figure 5:**
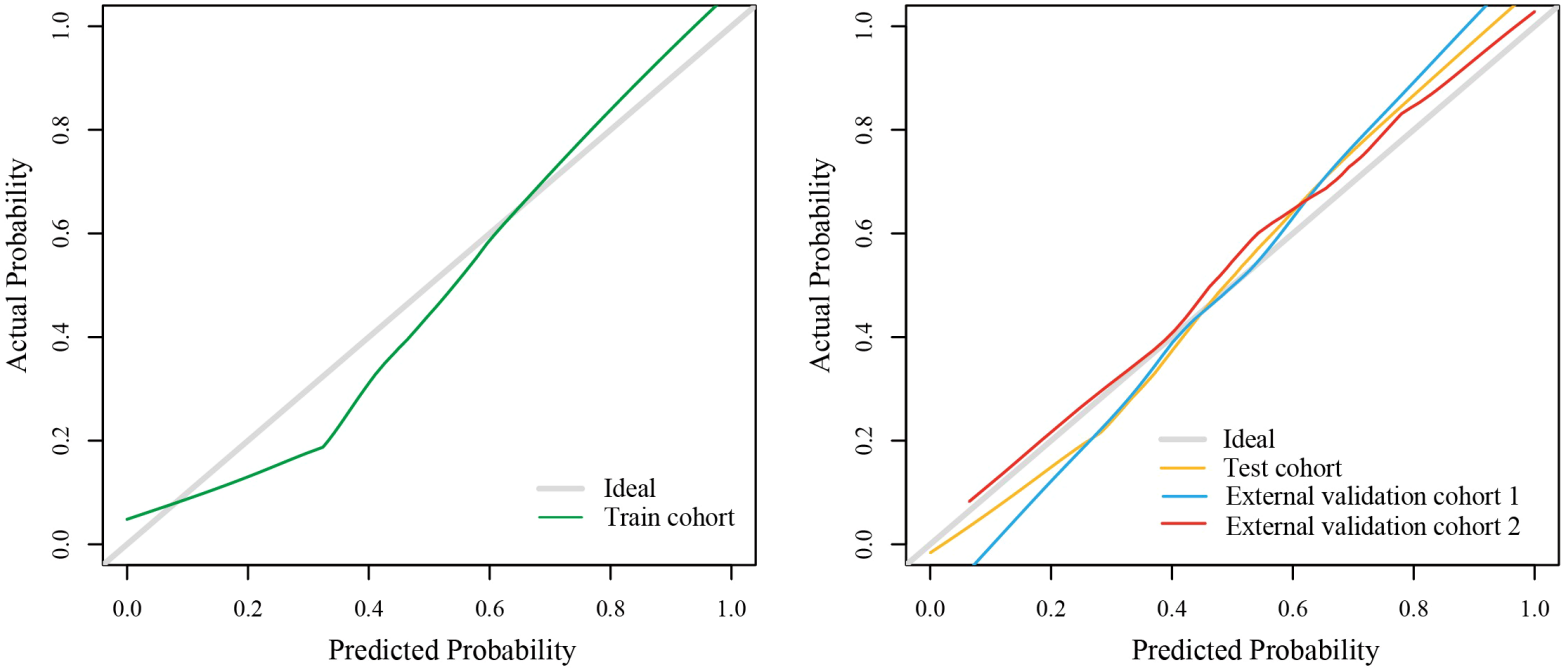
Calibration curves of the CIFM on the training cohort, test cohort, and two external validation cohorts.

**Figure 6:**
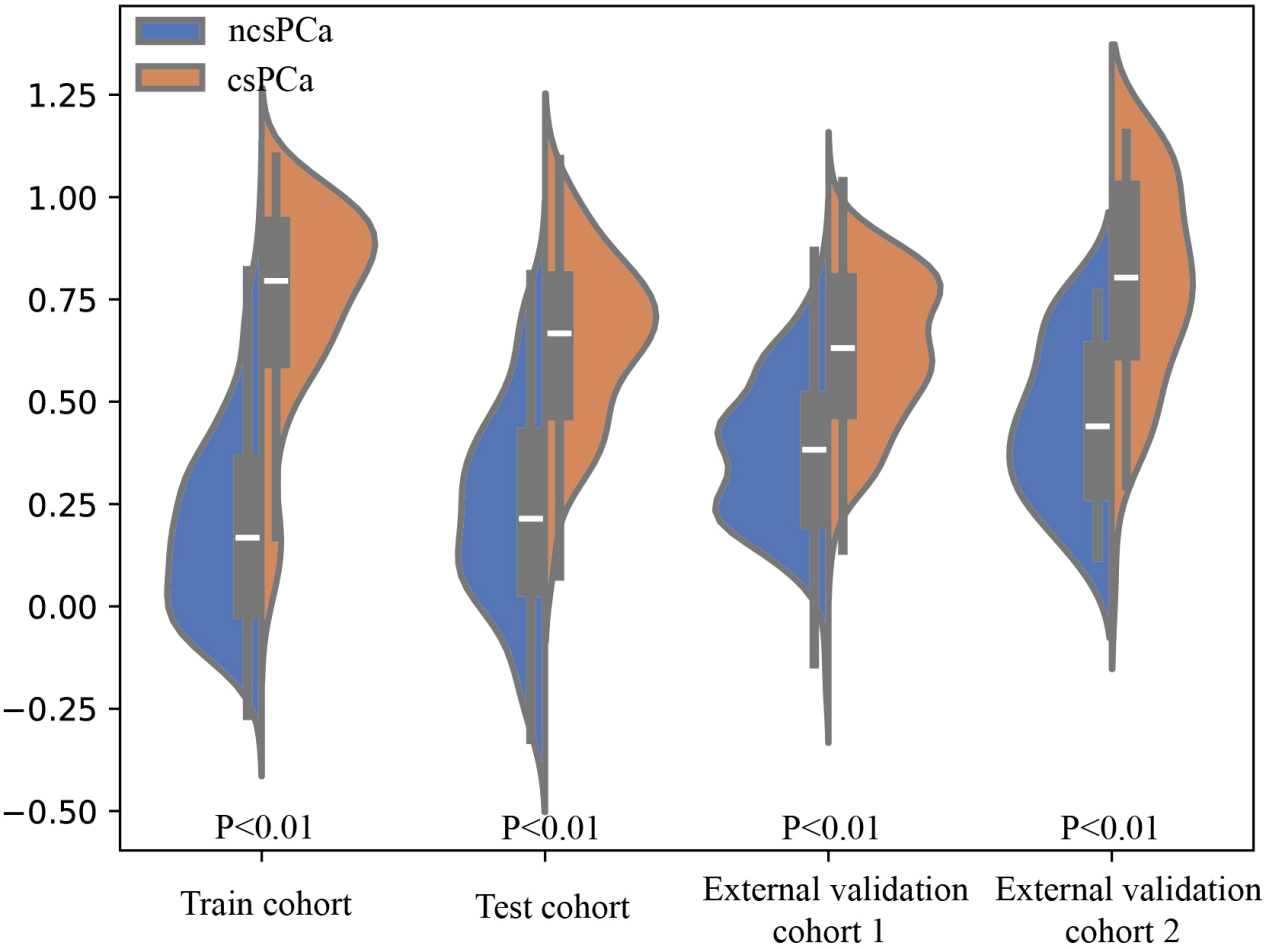
The score charts illustrate the ncsPCa and csPCa cases of the five centers evaluated by the CIFM.

The results of IDI and NRI analyses (**Supplementary Table 4**) demonstrated that CIFM achieved significant improvements over the clinical model across all datasets, indicating a marked enhancement in discriminating csPCa from ncsPCa patients. Similarly, compared with RTLM, CIFM showed positive improvements across datasets, suggesting improved classification performance. DCA results (**Figure 4**) further showed that CIFM provided greater net clinical benefit over a relatively broad range of threshold probabilities. In addition, calibration curves for CIFM in the training cohort, test cohort, and external validation cohorts (**Figure 5**) showed good agreement between predicted probabilities and observed outcomes, confirming the reliability of the model. **Figure 6** presents the overall distribution of predicted scores for the two classes, showing significant differences between positive and negative cases across all four cohorts (p < 0.01).

In addition, to further evaluate the applicability of the CIFM model in a different oncological imaging task, we constructed a CIFM model by integrating clinical features and deep imaging features in the NSCLC dataset and compared it with CM and RTLM. As shown in **Supplementary Table 3**, CIFM achieved higher AUC and overall diagnostic metrics than CM and RTLM in this NSCLC PD-L1 prediction task. This cross-task analysis was used as supplementary methodological validation of transferability across oncological imaging tasks and was not intended to indicate a direct disease-specific clinical association between NSCLC PD-L1 prediction and prostate cancer risk assessment.

### 3.4 Interpretability analysis of CIFM

**Figure 7:**
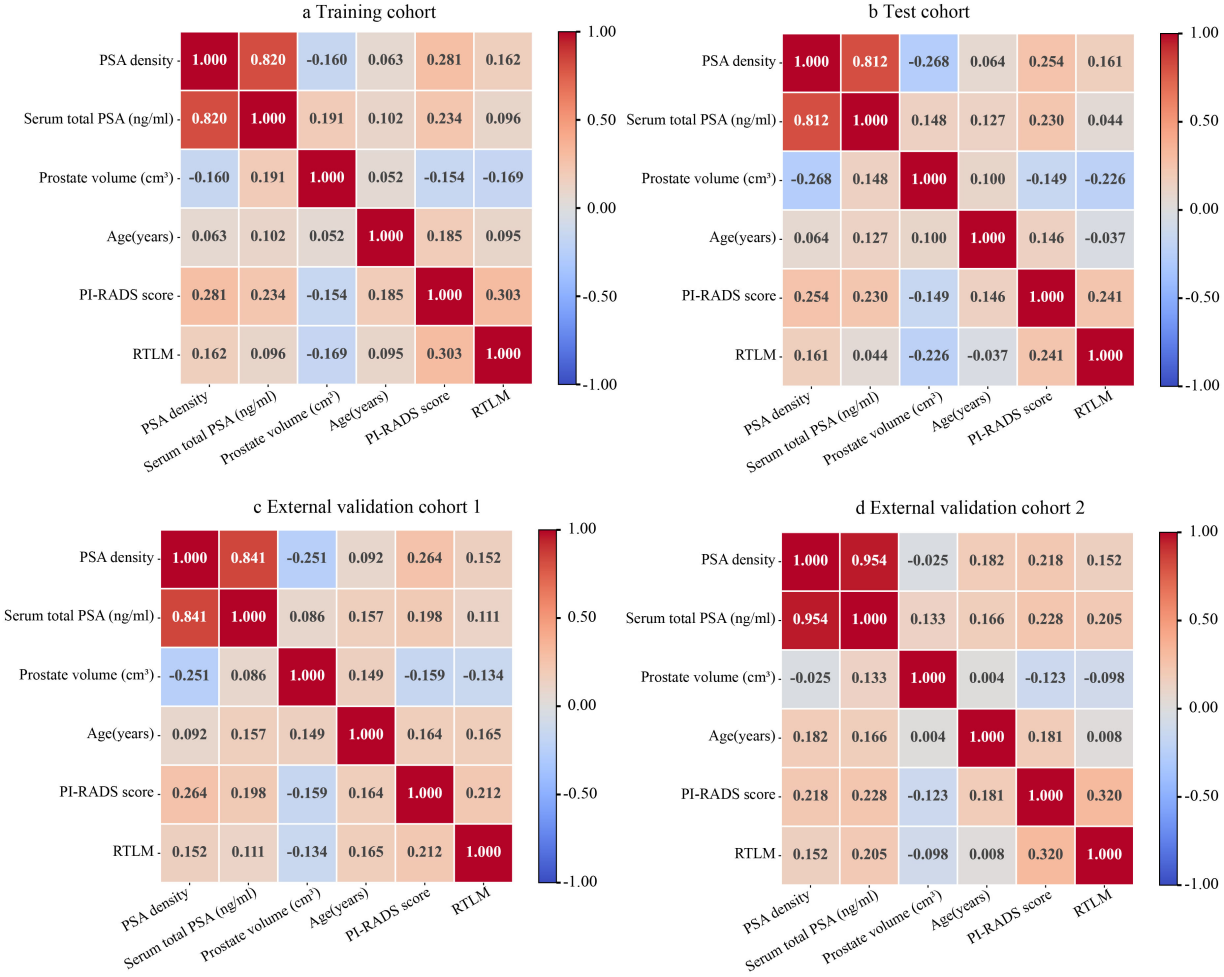
Pearson correlation heatmaps of the clinical variables and the RTLM-derived imaging representation used in the CIFM across the four cohorts. **(A)** Training cohort; **(B)** test cohort; **(C)** external validation cohort 1; and **(D)** external validation cohort 2. Red indicates positive correlations, blue indicates negative correlations, and the values in the cells represent Pearson correlation coefficients.

To examine the relationship between clinical variables and imaging-derived features, Pearson correlation coefficients were calculated and visualized as a correlation heatmap (**Figure 7**). Most clinical variables showed weak to moderate correlations with the imaging-derived features, suggesting that the two feature types were not highly redundant. This indicates that clinical variables and deep learning features may provide complementary information for CIFM: clinical variables reflect patient-level risk and structured clinical assessment, whereas imaging-derived features capture sub-visual patterns related to tumor heterogeneity. Therefore, the correlation analysis supports the rationale for multimodal fusion, although it should be interpreted as evidence of linear association and feature complementarity rather than causal interaction.

**Figure 8:**
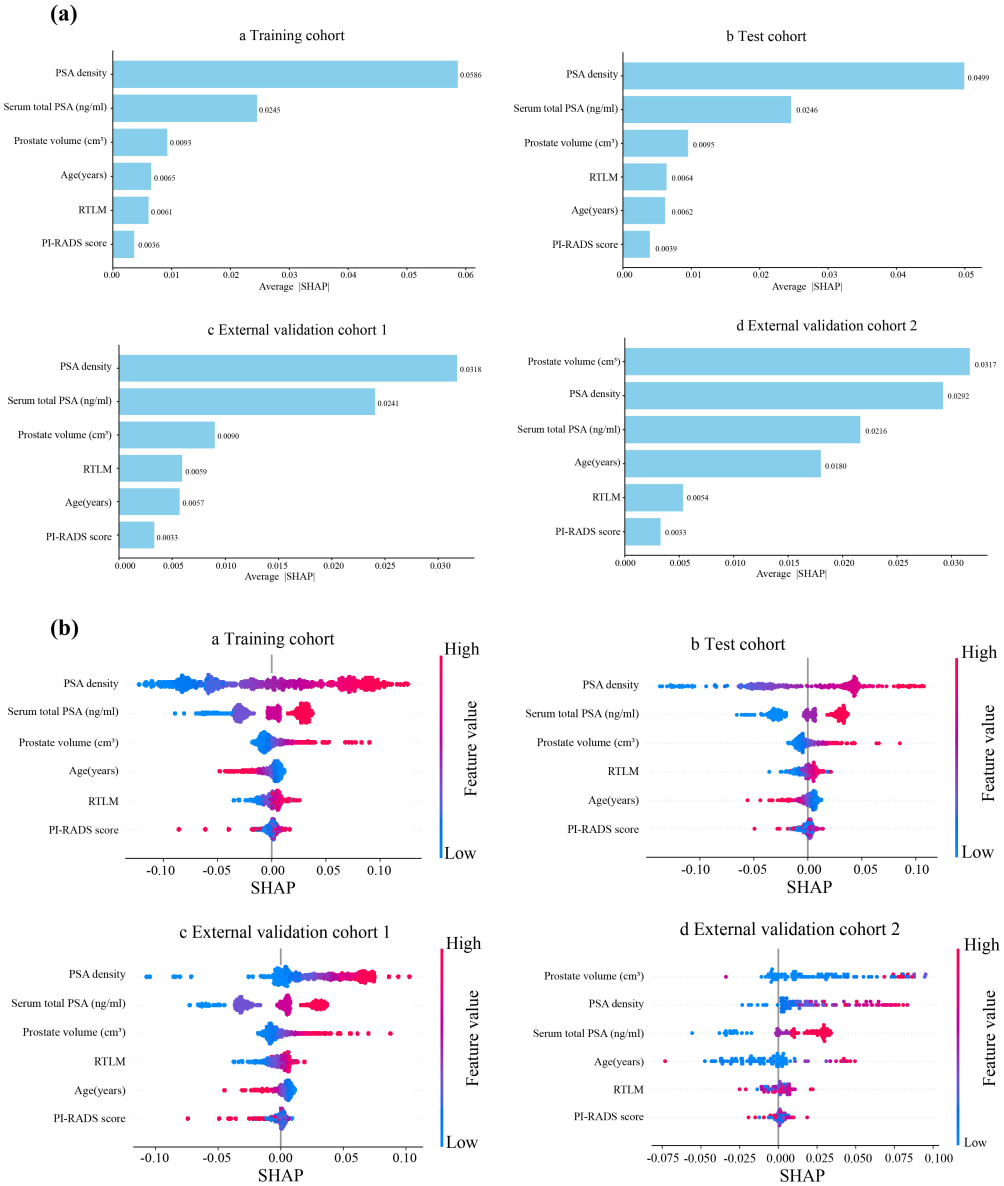
SHAP-based interpretability analysis of CIFM. **(A)** feature importance ranking plot based on mean absolute SHAP values, quantifying the relative contribution of each feature; **(B)** SHAP summary plot showing the direction and magnitude of each feature’s contribution to model prediction.

In addition, SHAP analysis was used to quantify the marginal contribution and directional effect of each input feature in CIFM. As shown in **Figure 8A**, PSA density and serum total PSA showed relatively high mean absolute SHAP values across the training cohort and validation cohorts, indicating that these clinically established risk indicators provided important patient-level information in the fusion model. However, this result should not be interpreted as evidence that CIFM was mainly driven by clinical variables alone. In the model comparison, RTLM consistently outperformed the clinical model, demonstrating the independent discriminative value of the RTLM-derived imaging representation. In CIFM, the RTLM output was incorporated as a compact imaging-derived representation, and its SHAP value reflects its marginal contribution after fusion with clinical variables rather than the full contribution of the underlying deep imaging features.

The SHAP summary plots in **Figure 8B** further showed that higher PSA density and higher serum total PSA generally shifted the model output toward higher predicted csPCa risk, which is consistent with clinical knowledge. Taken together, these findings suggest that RTLM provides the core imaging-based discriminative information, while clinical variables further refine patient-level risk estimation. Therefore, the improved performance of CIFM may be attributed to the integration of robust RTLM-derived imaging features with clinically meaningful risk indicators.

### 3.5 Ablation study of RTLM

To further validate the contribution of each component in RTLM, we conducted an ablation study, and the experimental results are shown in **Table 3**. When only the ResNet18 model was trained, the AUC values on the training and testing sets were 0.759 and 0.709, respectively. After introducing DINOv2-based fixed feature matching, the AUC values increased to 0.786 and 0.715, respectively, indicating that feature matching can extract useful transferable information from the vision foundation model. When the layer-wise weighting module alone or the channel-wise weighting module alone was added on the basis of feature matching, the testing AUC reached 0.740 and 0.742, respectively. The complete RTLM achieved the best performance, with AUC values of 0.894 and 0.848 on the training and testing sets, respectively. These results indicate that feature matching, layer-wise weighting, and channel-wise weighting all make positive contributions to model performance and can effectively alleviate the impact of negative transfer on model performance.

**Table 3:** Ablation study results.

Method	DINOV2	Feature matching	Layer weight	Channel weight	AUC	
					Train	Test
Baseline ResNet18	×	×	×	×	0.759	0.709
Fixed Feature Matching	√	√	×	×	0.786	0.715
Only Layer Weight	√	√	√	×	0.802	0.740
Only Channel Weight	√	√	×	√	0.812	0.742
RTLM	√	√	√	√	0.894	0.848

## 4 Discussion

In this study, we developed and validated the CIFM that integrates clinical features with deep imaging features for risk prediction of csPCa. The proposed model demonstrated favorable discriminative performance and showed consistent performance across the included multicenter clinical datasets. These results suggest that multimodal information may improve the model’s ability to characterize tumor heterogeneity and may provide supportive information for csPCa risk assessment.

The clinical variables identified through univariate analysis—age, serum total PSA level, PSA density, prostate volume, and PI-RADS score—were all significantly associated with the occurrence of csPCa, which is consistent with previous studies (27, 28). These features are well-established indicators for disease risk assessment. The CM constructed based on these variables achieved relatively stable predictive performance in both the training cohort and multicenter validation cohorts (AUC range: 0.726-0.758), supporting the clinical relevance of experience-based information in csPCa risk assessment. However, clinical variables are typically abstracted from physicians’ expertise and may overlook certain aspects of tumor spatial heterogeneity and imaging structural characteristics, thereby limiting further performance improvement. Consequently, models relying solely on clinical indicators may be insufficient for more accurate individualized risk prediction.

To address the tendency of conventional imaging models to overfit and exhibit limited generalization under small-sample conditions, this study proposed the RTLM. RTLM leverages the general feature representation capability of a vision foundation model (DINOv2) and employs feature-matching transfer networks to adaptively select task-relevant visual knowledge and determine the appropriate CNN layers and degree of knowledge transfer. This strategy may enhance the feature representation capability of the CNN, increase attention to critical features in prostate MRI, and reduce performance degradation caused by negative transfer. Across the included datasets, RTLM outperformed the clinical model in this study, suggesting improved discriminative performance under the current experimental setting. Moreover, RTLM showed favorable performance in cross-task validation on PD-L1 expression prediction in patients with NSCLC. This result should be interpreted as supplementary methodological evidence for transferability across oncological imaging tasks, rather than as direct evidence of clinical relevance to prostate cancer decision-making.

Building upon RTLM, this study further developed the CIFM by jointly modeling the DLS and key clinical variables. Compared with the clinical model alone or the imaging-based RTLM, CIFM integrates imaging and clinical information in a single prediction framework. This multimodal fusion improved the identification of high-risk csPCa patients in the included datasets and may support more consistent prediction across different cohorts. The performance gain of CIFM may be attributed to several factors. First, deep imaging features capture local structural characteristics and morphological heterogeneity of tumors, while clinical variables reflect patients’ overall pathological and physiological status; their integration provides a broader risk representation. Second, by integrating DLS with clinical variables, CIFM incorporates predictive information related to both imaging phenotype and clinical risk, thereby contributing to the observed performance improvement in this retrospective multicenter analysis.

Nevertheless, this study has several limitations. First, although multiple datasets were included and external validation was performed to evaluate the performance of the model across different cohorts, the retrospective nature of data collection may introduce selection bias, as patient inclusion could be influenced by data availability, image quality, biopsy availability, and center-specific clinical workflows. Second, heterogeneity in imaging acquisition protocols, scanner parameters, equipment, and clinical practices across different centers may affect image feature extraction and model performance. Although prostate MRI examinations were re-evaluated according to a unified PI-RADS v2.1-based standard and external validation was performed in independent cohorts, these measures cannot fully eliminate the influence of multicenter heterogeneity. Third, the ROI delineation strategy in this study mainly focused on the whole prostate and adjacent surrounding tissues rather than precise lesion-level segmentation. This strategy may reduce dependence on subjective lesion delineation and preserve global prostate information, but it may also dilute subtle lesion-specific differences. Finally, prospective validation has not yet been conducted, which limits the immediate clinical applicability of the proposed model. Future studies should include larger prospective multicenter cohorts and further compare whole-prostate, lesion-level, and hybrid ROI strategies to evaluate model performance and clinical applicability.

## Data Availability

The raw data supporting the conclusions of this article will be made available by the authors, without undue reservation.
