# Supplementary material for: Clinical-imaging fusion model for risk assessment of clinically significant prostate cancer
Source: Front Oncol. 2026 Jul 20;16:1844810. doi: 10.3389/fonc.2026.1844810 (PMC13429430; doi:10.3389/fonc.2026.1844810)
Supplement: Supplementary file 1 [file Table1.docx]

**Supplementary materials 1：Robust Transfer Learning Model based on Large Models**

DINOv2-Base (86 million parameters) is a vision foundation model trained on large-scale datasets and exhibits strong capability in representing general knowledge, which enables it to maintain high stability when recognizing diverse images. However, when such general knowledge is used to enhance the feature representation of convolutional neural networks (CNNs), negative transfer may occur, as not all generic knowledge is suitable for a given local task. To address this issue, we introduce a feature-matching–based transfer learning approach that can adaptively determine which knowledge should be transferred from the vision foundation model to the CNN and to which layers it should be transferred. This approach constructs two feature-matching transfer networks: one is used to identify the layer-wise correspondence between the vision foundation model and the CNN, and the other is used to select the specific features to be transferred as well as the degree of transfer. Through these networks, the feature learning process of the local CNN is effectively constrained and guided, enabling personalized knowledge transfer tailored to local data characteristics.

According to Equation (1), the difference between the feature representations of the vision foundation model and the CNN on local data is measured. By minimizing the L_2_ loss, the learning process of the CNN from the vision foundation model is constrained:

${{\text{L}_{\text{2}}\text{=}\text{||}\text{r}}_{\text{θ}_{\text{i}}}\text{(}\text{T}_{\text{θ}_{\text{i}}}^{\text{n}}\text{(}\text{x}\text{))-}\text{S}^{\text{m}}\text{(}\text{x}\text{)||}}_{\text{2}}^{\text{2}}$ $\text{(1)}$

where (m,n)∈α and α denotes a predefined set of matching pairs. $\text{S}^{\text{m}}\text{(}\text{x}\text{)}$ represents the intermediate feature map of the mmm-th layer of the vision foundation model, $\text{T}_{\text{θ}_{\text{i}}}^{\text{n}}\text{(}\text{x}\text{)}$ denotes the intermediate feature map of the nnn-th layer of the CNN, and $\text{r}_{\text{θ}_{\text{i}}}$​ is a linear transformation parameterized by $\text{θ}_{\text{i}}$​, which ensures consistency in the number of feature channels between the two models.

Each convolutional layer of a CNN consists of multiple convolutional kernels. The CNN extracts data features through these kernels, with each kernel generating an intermediate feature map. After linear transformation, these intermediate feature maps are individually matched with the intermediate feature maps generated by the transformer blocks of the vision foundation model, forming feature transfer channels. A feature-matching transfer network *f* is used to compute the weight $\text{w}_{\text{c}}^{\text{m}\text{,}\text{n}}$ for each feature transfer channel. The structure of the feature-matching transfer network *f* consists of P groups of fully connected layers, pooling layers, and a softmax layer. This network takes features from the vision foundation model as input and produces outputs through the softmax layer. As shown in Equation (2), $\text{w}_{\text{c}}^{\text{m}\text{,}\text{n}}$ is a learnable parameter that facilitates matching between the m-th layer features of the vision foundation model and the n-th layer features of the CNN:

$\text{w}_{\text{c}}^{\text{m}\text{,}\text{n}}\text{=}\text{f}\text{(}\text{S}^{\text{m}}\text{(}\text{x}\text{))}$ $\text{(2)}$

Subsequently, another feature-matching transfer network *g* is used to compute the layer-matching weight $\text{λ}_{\text{i}}^{\text{m}\text{,}\text{n}}$. The network *g* is composed of P groups of fully connected layers, pooling layers, and a ReLU6 activation layer, forming a fully connected network. It takes the features of the vision foundation model as input and produces outputs through the ReLU6 activation:

$\text{λ}_{\text{i}}^{\text{m}\text{,}\text{n}}\text{=}\text{g}\text{(}\text{S}^{\text{m}}\text{(}\text{x}\text{))}$ $\text{(3)}$

where $\text{λ}_{\text{i}}^{\text{m}\text{,}\text{n}}$>0. The parameters of the feature-matching transfer networks $\text{ϕ}_{\text{i}}$, consisting of both *f* and *g*, are updated based on changes in the CNN loss.

Finally, CNN parameters exhibit redundancy, and existing studies have demonstrated the feasibility of achieving both robustness and generalization simultaneously. The feature-matching transfer networks compute the adaptability of model layers between the vision foundation model and the local model, reflecting both the amount of general knowledge learned by each local model layer from the vision foundation model and the importance of each layer’s robustness. By combining Equations (1), (2), and (3), the overall knowledge transfer loss can be formulated as:

$\text{L}_{\text{wfm}}^{\text{m}\text{,}\text{n}}\text{(}\text{θ}_{\text{i}}\text{|}\text{x}\text{,}\text{ϕ}_{\text{i}}\text{)=}\sum_{\text{(}\text{m}\text{,}\text{n}\text{∈}\text{σ}\text{)}} \text{λ}_{\text{i}}^{\text{m}\text{,}\text{n}}\frac{\text{1}}{\text{HW}}\sum_{\text{c}} \text{w}_{\text{c}}^{\text{m}\text{,}\text{n}}\sum_{\text{k}\text{,}\text{u}} {\text{(}{\text{r}_{\text{θ}_{\text{i}}}\text{(}\text{T}_{\text{θ}_{\text{i}}}^{\text{n}}\text{(}\text{x}\text{))}}_{\text{c}\text{,}\text{k}\text{,}\text{u}}\text{-}{\text{S}^{\text{m}}\text{(}\text{x}\text{)}}_{\text{c}\text{,}\text{k}\text{,}\text{u}}\text{)}}^{\text{2}}\text{ }\text{ }$ $\text{(4)}$

where H×W denotes the spatial size of the output feature maps from $\text{T}_{\text{θ}_{\text{i}}}^{\text{n}}\text{(x)}$ and $\text{S}^{\text{m}}\text{(}\text{x}\text{)}$.

Accordingly, the total training loss in the first stage is defined as shown in Equation (5), where $\text{L}_{\text{org}}\text{(}\text{θ}_{\text{i}}\text{|}\text{x}\text{,}\text{y}\text{)}$ denotes the cross-entropy loss of the local model *T_i_*, and $\text{η}$>0 is a hyperparameter:

$\text{L}_{\text{todal}}\text{(}\text{θ}_{\text{i}}\text{|}\text{x}\text{,}\text{y}\text{,}\text{ϕ}_{\text{i}}\text{,}\text{θ}_{\text{global}}\text{)=}\text{L}_{\text{org}}\text{(}\text{θ}_{\text{i}}\text{|}\text{x}\text{,}\text{y}\text{)+}\text{η}\text{L}_{\text{wfm}}\text{(}\text{θ}_{\text{i}}\text{|}\text{x}\text{,}\text{ϕ}_{\text{i}}\text{)}$ $\text{(5)}$

The optimization objective of the local model *T_i_* in the first stage is:

$\text{min}_{\text{θ}_{\text{i}}}\text{E}_{\text{(}\text{x}\text{,}\text{y}\text{)\textasciitilde}\text{D}_{\text{i}}}\text{(}\text{L}_{\text{todal}}\text{(}\text{θ}_{\text{i}}\text{|}\text{x}\text{,}\text{y}\text{,}\text{ϕ}_{\text{i}}\text{,}\text{θ}_{\text{global}}\text{))}$ $\text{(6)}$

After completing the transfer learning stage, features are extracted from the CNN model to construct the deep learning signature (DLS).

Table S1 Distribution of PD-L1 patients

|  | Training cohort (n=74) | | | Validation cohort (n=45) | | |
| --- | --- | --- | --- | --- | --- | --- |
|  | PD-L1 <1%  (n=34) | PD-L1 >1%  (n=40) | *P*-Value | PD-L1 <1%  (n=18) | PD-L1 >1%  (n=27) | *P*-Value |
| Age (mean ± SD, years) | 62.47±10.03 | 63.30±12.95 | 0.762 | 61.39±8.23 | 63.33±11.28 | 0.534 |
| Sex | | | | | | |
| Male | 18 | 28 | 0.132 | 10 | 24 | 0.011 |
| Female | 16 | 12 |  | 8 | 3 |  |
| Pathological type | | | | | | |
| Squamous cell carcinoma | 7 | 0 | 0.003 | 1 | 16 | <0.001 |
| Adenocarcinoma | 27 | 36 |  | 17 | 11 |  |
| Non-squamous non- adenocarcinoma | 0 | 4 |  | 0 | 0 |  |
| Staging | | | | | | |
| I | 16 | 9 | 0.006 | 11 | 7 | 0.012 |
| II | 7 | 2 |  | 2 | 11 |  |
| III | 4 | 9 |  | 0 | 5 |  |
| IV | 7 | 20 |  | 5 | 4 |  |

Table S2 Clinical model parameters

| IW | | | |
| --- | --- | --- | --- |
| 0.0947633071873197 | 0.890070197657577 | -0.456312165503862 | 0.903827936814369 |
| -0.646002671699510 | -0.0339353039239720 | 0.0555054354495042 | 0.274205139480356 |
| 0.944151869190882 | -0.966366880173070 | 0.473976296940675 | -0.512164044760431 |
| 0.562606493357896 | 0.623319416782579 | 0.556567751155197 | -0.561243124187902 |
| -0.373865739670554 | -0.449765064643756 | -0.147349239223348 | 0.0985039170361157 |
| -0.160049567441602 | 0.861147555228313 | 0.368503205305905 | 0.145613980100522 |
| B | | | |
| 0.181241513348071 |  |  |  |
| 0.286748701960290 |  |  |  |
| 0.611006523561143 |  |  |  |
| 0.903293004808808 |  |  |  |
| 0.259514535984739 |  |  |  |
| 0.491188033043848 |  |  |  |
| B0 | | | |
| 0 |  |  |  |
| TF | | | |
| sigmoid |  |  |  |
| LW | | | |
| 0 |  |  |  |
| 0.946720565803682 |  |  |  |
| 0 |  |  |  |
| 0 |  |  |  |
| 0 |  |  |  |
| 1.03455844106336e-05 |  |  |  |

Notes：IW: Weights from each input feature to each hidden-layer node; B: Bias term for each hidden-layer node; B0: Output-layer intercept; TF: Hidden-layer activation function; LW: Weights from hidden layer to output score.

Table S3 Model diagnostic performance

| **Model** | **Sets** | **AUC**  **(95% CI)** | **Sensitivity** | **Specificity** | **Accuracy** | **PPV** | **NPV** |
| --- | --- | --- | --- | --- | --- | --- | --- |
| CM | Training cohort | 0.712  (0.595-0.829) | 0.525  (21/40) | 0.882  (30/34) | 0.689  (51/74) | 0.840  (21/25) | 0.612  (30/49) |
|  | Test cohort | 0.683  (0.505-0.861) | 0.222  (6/27) | 0.833  (15/18) | 0.467  (21/45) | 0.667  (6/9) | 0.417  (15/36) |
| RTLM | Training cohort | 0.892  (0.816-0.967) | 0.900  (36/40) | 0.824  (28/34) | 0.865  (64/74) | 0.857  (36/42) | 0.875  (28/32) |
|  | Test cohort | 0.877  (0.771-0.982) | 0.815  (22/27) | 0.833  (15/18) | 0.822  (37/45) | 0.880  (22/25) | 0.750  (15/20) |
| CIFM | Training cohort | 0.908  (0.842-0.975) | 0.950  (38/40) | 0.735  (25/34) | 0.851  (63/74) | 0.809  (38/47) | 0.926  (25/27) |
|  | Test cohort | 0.918  (0.829-1.000) | 0.852  (23/27) | 0.833  (15/18) | 0.844  (38/45) | 0.885  (23/26) | 0.789  (15/19) |

Table S4 Model evaluation improvement sheet

|  | IDI | | NRI | |
| --- | --- | --- | --- | --- |
| Cohorts | Model2 | Model1  (Clinical model) | Model2 | Model1  (Clinical model) |
| Training | CIFM | 0.3804(P<0.00001) | CIFM | 1.2988(P<0.00001) |
| Test | CIFM | 0.2893(P<0.00001) | CIFM | 0.8973(P<0.00001) |
| External validation  Cohort 1 | CIFM | 0.1765(P=0.00108) | CIFM | 0.8202(P<0.00001) |
| External validation  Cohort 2 | CIFM | 0.1802(P=0.00011) | CIFM | 0.9848(P<0.00001) |
|  | Model2 | Model1(RTLM) | Model2 | Model1(RTLM) |
| Training | CIFM | 0.0745(P<0.00001) | CIFM | 0.7798(P<0.00001) |
| Test | CIFM | 0.1555(P<0.00001) | CIFM | 1.0240(P<0.00001) |
| External validation  Cohort 1 | CIFM | 0.0760(P=0.00001) | CIFM | 0.5159(P<0.00001) |
| External validation  Cohort 2 | CIFM | 0.0985(P=0.04837) | CIFM | 0.4589(P=0.02923) |
|  | Model2 | Model1(Z-SSMNet) | Model2 | Model1(Z-SSMNet) |
| Train | CIFM | 0.3033(P=0.00000) | CIFM | 0.9383(P=0.00000) |
| Test | CIFM | 0.3544(P=0.00000) | CIFM | 1.0634(P=0.00000) |
| External validation  Cohort 1 | CIFM | 0.2816(P=0.00000) | CIFM | 0.9343(P=0.00000) |
| External validation  Cohort 2 | CIFM | 0.1952(P=0.00141) | CIFM | 0.6797(P=0.00146) |
|  | Model2 | Model1(PICG2) | Model2 | Model1(PICG2) |
| Train | CIFM | 0.3417(P=0.00000) | CIFM | 1.0138(P=0.00000) |
| Test | CIFM | 0.3637(P=0.00000) | CIFM | 1.1229(P=0.00000) |
| External validation  Cohort 1 | CIFM | 0.2899(P=0.00000) | CIFM | 0.9041(P=0.00000) |
| External validation  Cohort 2 | CIFM | 0.2112(P=0.00032) | CIFM | 0.7511(P=0.00034) |

Notes**:** The NRI values were calculated using the continuous NRI approach. No predefined risk-category threshold was applied.

Table S5 Comparison of the number of parameters of different models

| **Model** | **Parameters** | **Parameters (M)** |
| --- | --- | --- |
| CM | 29 | 0.000029 |
| RTLM | 14,994,465 | 14.994465 |
| CIFM | 14,994,618 | 14.994618 |

**Notes:** For RTLM, the parameter count refers to the trainable ResNet18-based local model and transfer-related modules used in the target task. The DINOv2-Base vision foundation model (86M parameters) was used as a feature-guidance model rather than as a directly fine-tuned end-to-end predictor. For CIFM, the count includes the RTLM-derived imaging representation and the parameters of the clinical-imaging fusion classifier.

Table S6. Inter-reader agreement for PI-RADS assessment between two radiologists

| **Variable** | **Categories** | **Weighting method** | **Weighted κ** | **P value** |
| --- | --- | --- | --- | --- |
| PI-RADS score | 1–5 | Quadratic weights | 0.964 | <0.001 |

**Note:** Inter-reader agreement between the two radiologists was assessed using weighted Cohen’s kappa. Because PI-RADS score is an ordinal variable, quadratic weights were applied. κ, kappa coefficient.
